# Research on the Relationship Between Safety Leadership, Safety Attitude and Safety Citizenship Behavior of Railway Employees

**DOI:** 10.3390/ijerph17061864

**Published:** 2020-03-13

**Authors:** Mengjie Li, Huaiyuan Zhai, Junjie Zhang, Xiangcheng Meng

**Affiliations:** 1 School of Economics and Management, Beijing Jiaotong University, Beijing 100044, China; 19113055@bjtu.edu.cn (M.L.); 18120646@bjtu.edu.cn (J.Z.); 2 School of Economics and Management, Beijing Jiaotong University Haibin College, Cangzhou 061199, Hebei, China; 3 School of System Engineering and Engineering Management, City University of Hong Kong, Hong Kong, China; xcmeng3-c@my.cityu.edu.hk

**Keywords:** safety leadership, safety attitude, safety citizenship behavior, structural equation model

## Abstract

The daily operation and maintenance work of railways are very dangerous. Railway employees often have safety accidents while working, and the safety citizenship behavior (SCB) of railway employees can effectively reduce the accident rate. Therefore, it is of great significance to identify the main safety constructs affecting the SCB of railway employees to minimize accidents. This paper puts forward a supposed model of the influence mechanism of safety leadership (SL) on employee SCB through the mediation of safety attitude (SA). A questionnaire was used with railway employees, and 238 valid responses were finally collected. A structural equation model (SEM) was used to explore the relationship between SL, SA and SCB. The results showed the SL is positively related to the SA, and it can further promote the SCB of railway employees. In addition, SA has a positive impact on employee SCB.

## 1. Introduction

At present, with the vigorous development of freight industry, the important role of railway transportation is becoming more and more prominent. However, risk still exists during the daily operation and maintenance of railways, and railway employees often have safety accidents at work [1]. For example, a Japanese express train derailed in Nagasaki and caused six carriages to derail in July 2003 due to the unsafe behavior of workers, and more than 60 people were injured or died. Also, an accident occurred with the Chinese train D301 from Beijing South Station to Fuzhou South Station due to the irregular operation of workers, leading to 172 injuries and 40 fatalities [2]. In 2015, there were 36 accidents and 39 deaths of employees [3]; in 2016, there were 24 accidents and 27 deaths of employees in the China Railway Corporation [4]; in 2017, there were 18 accidents and 24 deaths of employees in the China Railway Corporation [5]. From the data of 2015-2017, the safety situation of railway employees in China is still not optimistic, and the number of accidents and deaths is still very high.

Based on the previous studies of scholars, accidents can be avoided by improving safety citizenship behavior (SCB) of the employees, which is very significant to reduce violations by the organization [6,7].

Hofmann et al. [8,9] formally put forward the concept of SCB for the first time, namely, the voluntary behavior of construction personnel to ensure the safe performance of other team members and achieve the safety goals of the project and organization. It was found that SCB is very important for improving the safety performance of working groups and emphasizing mutual support among employees, so as to improve organizational efficiency. On this basis, Shama et al. [10] further defined SCB as the behavior of helping other group members to improve safety spontaneously outside of work, which was further considered an important participating factor in the safety of working groups [11,12]. Unsafe acts contribute predominantly to construction accidents, and increasing safety behavior among groups is essential to reduce accidents [13,14]. Therefore, this study considered SCB, which has a positive effect on reducing the number of safety accidents.

Safety leadership (SL) is also considered a construct that can significantly influence work safety. SL refers to a process in which a person guides and influences other individuals or groups to achieve safety objectives when completing organizational tasks [15]. The person committed to SL and influencing others is called a safety leader [16]. SL success is mainly affected by the personal charisma and characteristics of leaders, and a safe atmosphere plays an intermediary role between SL and safety behavior [17]. Grill [18] thought direct and indirect leadership practices can influence the safety performance of workers at construction sites, and there are numerous SL interventions being deployed within the rail industry [19,20]. In addition, there is evidence that safety-specific transformational leadership positively impacts safety outcomes including safety behaviors [21].

In addition, safety attitude (SA) has proven to be effective in improving employee safety. Particularly, SA is a kind of psychological activity, and it is implicit, but it directly affects and dominates people’s behavior [22]. SA is considered the stable and general reflection of employees to work safely, which can help recognize the importance and facilitate the implementation of safety policies, and further promote the commitment to implement safety rules and regulations [23,24]. Monazzam and Soltanzadeh [25] found that if workers are overly optimistic that no accidents will happen to them, they may be more likely to suffer risks and end up with injuries. Rau [26] pointed out that SA has proven effective in predicting accidents in traffic and the workplace. Safety communication among railway workers is fundamental to effective safety management [27]. However, evidence suggests that poor safety communication is a common problem in railway workplaces [28].

Currently, there is no research focusing on the influencing mechanism of SCB with critical safety constructs such as safety attitude (SA) and safety leadership (SL). Therefore, it is of great significance to study the relationship among employees’ SL, SA and SCB, to find out the channels that improve employees’ SCB, and then to reduce the occurrence of safety accidents. To fill this research gap, this paper proposed a hypothetical model of SL’s influence on SCB through SA of railway workers. A questionnaire was used with railway employees in a sample of 254 participants, and the method of structural equation modelling was used to explore the relationship between SL, SA and SCB. This paper is the first and a meaningful study investigating the interrelationship between SA, SL and SCB in the rail industry. In many articles, SCB is researched in the chemical industry [12] or construction industry [13], which has a large turnover of personnel and an easily changing organizational structure. The railway employees proposed in this paper mainly refer to railway operation and maintenance personnel. These personnel have the following characteristics: the personnel distribution in different working groups is fixed and remains unchanged for a long time, and the direct leadership of each group is basically unchanged. This stable management organization structure is convenient to study the factors that influence safe citizenship behavior, and the conclusions are more representative.

## 2. Materials and Methods

### 2.1. Materials

#### 2.1.1. Safety Leadership and Safety Attitude

Hunter et al. [29] and others believed that leadership behavior and leadership trust have a positive impact on employee attitudes. Sokol [30] believed that the leadership of college lecturers plays a positive role in shaping the creative attitude of students. Moreover, a high sense of leadership can motivate followers to express their sense of responsibility more fully and make them act in a consistent way. Guay et al. [31] verified that transformational leadership has a significant impact on safety climate. Mullen [32] displayed that safety-specific transformational leadership was positively and significantly associated with safety compliance, safety participation and SA of the employees, which further affects the improvement of safety behaviors [33].

Therefore, the following hypothesis H1 was proposed:

**Hypothesis** **(H1).** SL has a positive impact on SA.

#### 2.1.2. Safety Attitude and Safe Citizenship Behavior

Gharibi et al. proposed that personal factors leading to unsafe behaviors include inappropriate attitudes and cognition, which affect people’s behaviors and the possibility of accident occurrence [34]. Hofmann et al. found that employee attitudes can effectively reduce the occurrence of accidents [8,9]. Jahangiri et al. [35] proposed that each dimension of SA has different mediating roles between safety management leadership and the effects of implementing a safety system. In terms of behavioral theory, Kao et al. [33] thought that workers’ SA and safety behaviors have a direct relationship. Research by Ledesma et al. [36] showed that SA is significantly related to behavioral safety, and especially, the attitude of employees to accident prevention significantly affects their behavioral safety at both individual and organizational levels [37]. Meng et al. [38] further proposed that increased attention to safety and attitude of workers play positive roles in promoting safety citizenship behavior (SCB).

Therefore, the following hypothesis H2 was proposed:

**Hypothesis** **(H2).** SA has a positive impact on SCB.

#### 2.1.3. Safe Leadership and Safe Citizenship Behavior

There is a significant relationship between leadership and behavioral safety in some high-risk industries [39,40]. O’dea and Flin [41] pointed out that senior managers can directly influence safety behaviors as well as the atmosphere and expectations of the organization and enterprise. Hackett et al. [42] and others believed that transformational leadership can promote organizational citizenship behavior. In addition, organizational citizenship behavior of leaders promotes organizational citizenship behavior of subordinates, which is verified as imitating the behavior of their superiors [43]. Therefore, leader–member exchange has a positive impact on organizational citizenship behavior [44]. In detail, leader–member exchange affects organizational citizenship behaviors in accordance with personal respect and support of direct superiors [45]. Also, as one of the particular kinds of organizational citizenship behavior, SCB was verified to be positively influenced by leadership–member exchange [46]. Strengthening the leadership and communication ability of safety personnel and creating a good safety atmosphere on site will help to improve safety performance [47]. Kapp [48] proposed that the safety compliance of an employee will be improved as the SL of the supervisor increases.

Therefore, the following hypothesis H3 was proposed:

**Hypothesis** **(H3).** SL has a positive impact on SCB.

**Figure 1:**
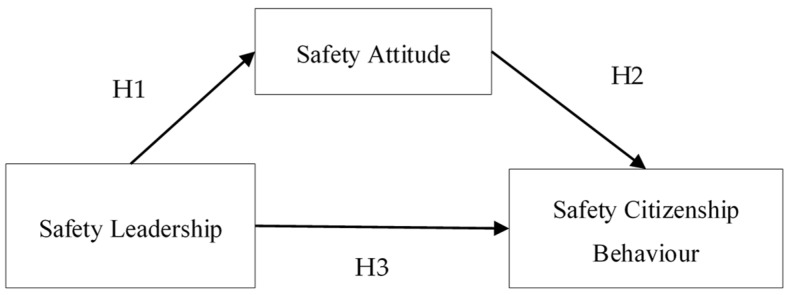
Framework of the influence model and the hypotheses among safety citizenship behavior (SCB), safety attitude (SA) and safety leadership (SL).

All hypotheses are shown in Figure 1. Taking railway employees as the research object, this paper uses structural equation modeling (SEM) to study the relationship between SL, SA and SCB and to find out the way to improve citizens’ safety behavior and reduce the occurrence of safety accidents.

### 2.2. Methods

#### 2.2.1. Dimension Division

While studying the relationship between leader–member exchange and SCB, Hofmann proposed that safety citizenship behavior (SCB) is a multidimensional construct including helping colleagues, safe proposal, responsibility awareness, safe communication, civic ethics and spontaneous change [8,9]. Curcuruto and Griffin [12] proposed a four-dimensional model of SCB according to the specific context of the chemical industry, namely safety stewardship, affective commitment, safety voice and psychological ownership. Liu et al. [49] further proposed a four-dimensional model of SCB: safety proposal, responsibility awareness, active participation and helping colleagues. For this study, after considering the complexity of the railway employees’ work site and implementation of the safety rules and regulations, this paper divides SCB into four dimensions in line with Meng et al. [38]: mutual aid among workers, relationship between superior and subordinate, participation in suggestion-making and self-control.

For safety attitude (SA), Sexton et al. and Loosemore and Malouf [50,51] believe that the SA scale consists of five dimensions: teamwork atmosphere, safety atmosphere, management concept, job satisfaction and working conditions. White et al. [52] proposed that SA should be classified as advantages (e.g., personal safety of self and co-workers), disadvantages (e.g., inconvenience to customer/clients and workload), referents (e.g., supervisors, work colleagues, customers), barriers (e.g., time and cost) and facilitators (e.g., training and knowledge, equipment availability) of safety adherence. Therefore, combined with the above views and the characteristics of railway employees, this paper divided the SA into three dimensions: safety awareness, safety behavior tendency and safety emotion.

In terms of safety leadership (SL), Lee et al. [53] believed that SL can be divided into three dimensions, namely empowerment leadership, knowledge sharing, and safety climate. Clarke [54] further divided the SL into supervisory monitoring and action. Stiles and Ryan [14] identified that SL interventions were influenced by five themes: context, preparation, communication, action, and leadership behavior and style. Lu and Yang [55] thought that SL should be measured in terms of three dimensions: security motivation, security policy and security concerns. Wu [56] further revised existing SL scales to three dimensions of safety coaching, safety caring and safety controlling. In view of the fact that employees in leading positions of railway work who dispatch personnel face problems of scattered working places and poor compliance of lower-level employees, this paper divided the SL into three dimensions: leadership behavior, safety concern and security control.

The dimensions and abbreviations of SCB, SL and SA are shown in Table 1 below.

#### 2.2.2. Questionnaire Design

The scale for assessing SCB included 26 items. An example item was “I will help other workers to ensure their safe work”, which was used to measure the performance of employees in terms of mutual help. The higher the score was, the more consistent the employee was with the description, and the better the SCB. In the questionnaire that was given to team leaders, the first person “I” was replaced by the third person “he” to ensure objectivity. The SL scale included 17 items that were designed from the perspective of employees. For example, “my leaders follow the safety rules by example” was used to measure the safety behavior of leaders. The higher the score was, the better the employee thought the leader was in terms of the safety, and the better the SL. To measure SA, the scale proposed here involved 19 items. For example, “I think on-site work is highly dangerous” was used to measure employee awareness of safety. The higher the score was, the better the SA of employees. All items were measured using an eight-point Likert scale. All items exhibited verbal anchors of “strongly disagree” and “strongly agree” at points 1 and 8, respectively. At the same time, in order to prevent the persons who were tested from answering the questions indiscriminately, reverse questions were set in the questionnaire, such as “work accidents are mostly due to bad luck” and “too much attention to safety procedures will affect work efficiency”. In terms of these items, the higher the score was, the worse the SA, SL and SCB of employees. Therefore, data were converted to revise the numbers. Please refer to Appendix A for details of these three questionnaires.

#### 2.2.3. Sample Collocation

For the sample size calculation, Bollen recommended a sample of 150 or more for covariance-based SEM [57], while an actual sample size of 254 was obtained in this study. The questionnaires were collected with the assistance of local labor unions. A total of 254 team leaders and team members were to conduct an onsite questionnaire survey in a railway station. Two hundred fifty-four SCB scale questionnaires were distributed to team leaders on site to evaluate the SCB of each team member, and 254 questionnaires consisting of SL scale, employee’s own SA scale and employee’s own SCB scale were distributed to each team member to evaluate the attitude and leadership of their direct leaders and personal SA and SCB. Questionnaires with a large number of missing items and blanks were removed, and questionnaires that were consistently filled out erroneously were abandoned. Finally, 238 valid questionnaires were obtained, with an effective recovery rate of 93.7%. For ethical considerations, all respondents provided their informed consent before participating in the study. This research was approved by the Internal Review Board (IRB) of Beijing Jiaotong University.

#### 2.2.4. Data Analysis

Data were analyzed after collection and collation were completed. SPSS 19.0 (IBM, Armonk, New York, USA) and AMOS 26.0 (IBM, Armonk, New York, NY, USA) were used for data processing and statistical analysis. The specific steps were as follows:

(1) KMO and Bartlett Sphericity test were used to test the data validity [58];
(2) the reliability of the scale was tested by Cronbach’s alpha [59,60];
(3) for each variable, the parameter significance, convergence validity and discriminant validity were analyzed;
(4) the hypotheses were tested using the structural equation modeling technique;
(5) specific improvements were considered based on the results of the data analysis.

## 3. Results

### 3.1. Data Validity

SPSS 19.0 was used to test the questionnaire. All variables were significant at the level of 0.05. KMO and Bartlett tests of sphericity were carried out. The results showed that the KMO value of the scale was 0.880, which was more than 0.7, and the sig value was less than 0.05, which proved that the data were valid and suitable for factor analysis [58].

### 3.2. Reliability Analysis

Cronbach’s coefficient was used to measure the reliability of the questionnaire. A Cronbach’s alpha value above 0.70 is recommended to ensure data reliability [61]. SPSS 19.0 was used to analyze the reliability of SL, SA and SCB. The results are shown in Table 2 below.

It can be seen from Table 2 that the Cronbach’s alphas of SL, SA and SCB were all greater than 0.8. The CITC of each measurement item of each construct was greater than 0.6; even nearly half of the were greater than 0.7, and the other half of the values were greater than 0.8. The alpha value of each measurement model was less than the alpha value of the initial measurement model after deleting each item. Therefore, the items of SL, SA and SCB had good reliability, namely 0.96 for those of SL, 0.902 for those of SA and 0.842 for those of SCB, which were all bigger than the Cronbach’s alpha value of the item deleted.

### 3.3. Significance Analysis, Convergence Validity Analysis and Discriminant Validity Analysis

AMOS 26.0 was used to analyze the significance of the parameters and convergence validity of each safety construct, as shown in Table 3.

It can be seen from Table 3 that the standardized factor load (Std) of each observation variable was greater than 0.7, the average variance extracted (AVE) of each latent variable was greater than 0.5, and the composite reliability (CR) was greater than 0.6, so it was verified to have high convergence validity. All factor loads were greater than 0.6, and all squared multiple correlations (SMCs) were greater than 0.5, indicating that the reliability of all subjects was good. All *P* values indicated the results were significant (Hair et al. [62]).

The discriminant validity test of each variable is shown in Table 4 below. It can be seen from Table 4 that the discriminant validities of SL and SCB were good. In contrast, the discriminant validity of SA was moderate but acceptable. Therefore, the discriminant validity of each variable was acceptable. From the perspective of SL, 0.9 is greater than 0.77 and 0.82, and the discriminant validities between SL and SCB, SL and SA were good. From the perspective of SCB, 0.86 was greater than 0.77 and 0.82, and the discriminant validities between SCB and SL, SCB and SA were good. From the perspective of SA, 0.80 is less than 0.82 but more than 0.78, the discriminant validity between SA and SL was moderate but acceptable, and the discriminant validity between SA and SCB was good. Therefore, the discriminant validity of each safety construct was verified to be acceptable [63].

### 3.4. Hypothesis Test

**Figure 2:**
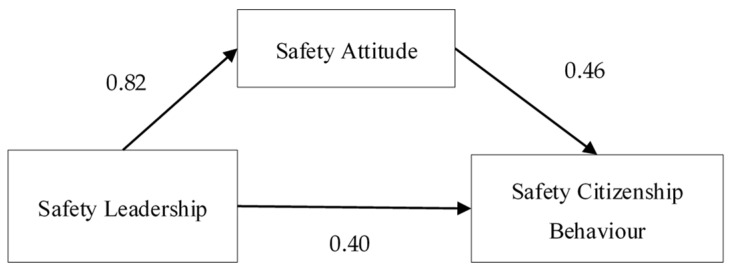
Simulated hypothesis model of SA, SL and SCB.

The above analysis showed the validity of the questionnaire data, which can be used to test the hypothesis proposed in this paper. AMOS 26.0 was used to build the structural equation model, and the results are shown in the following Figure 2 and Table 5. Figure 1 shows the quantitative results of the hypotheses and the influence among SA, SL and SCB. In addition, it can be seen from Table 5 that, except for the chi-square divided by degrees of freedom (χ^2^/df) (5.545) being slightly higher than the acceptable range of 3 to 5, the measurements were all in acceptable ranges: root-mean-square residual (RMR) was less than 0.05, root-mean-square error of approximation (RMSEA) was less than 0.05, parsimony goodness-of fit-index (PGFI) was greater than 0.5, incremental fit index (IFI) and comparative fit index (CFI) were greater than 0.9, and goodness-of-fit index (GFI) was greater than 0.8 and close to 0.9. This indicated that the fitness between the actual structural equation model and empirical data was good; therefore, the model can be used to analyze the interaction between different safety constructs [63].

**Figure 3:**
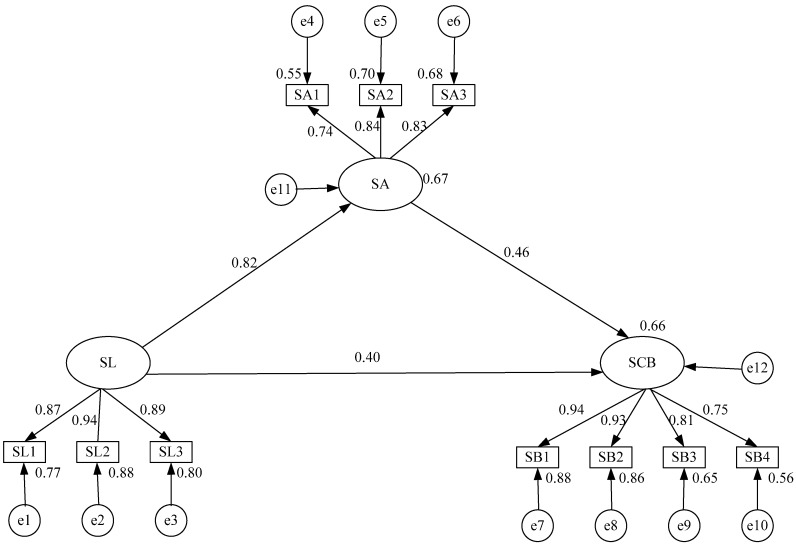
Structural equation model of safety leadership, safety attitude and safety citizenship behavior. SL is safety leadership; SA is safety attitude; SCB is safety citizenship behavior. SL1 is leadership behavior; SL2 is safety concerns; SL3 is security control; SA1 is safety awareness; SA2 is safety behavior tendency; SA3 is safety emotion; SB1 is mutual aid among workers; SB2 is relationship between superior and subordinate; SB3 is participation in suggestion-making; SB4 is self-control. e1 to e12 are residuals describing the part of an endogenous variable that cannot be explained. Values on the paths are the standardized path coefficients. Values next to rectangles and latent variables represent the variance the factor accounted for in the model.

The empirical results of the model are shown in Figure 3, and hypothesis validation is shown in Table 6.

In Figure 2, the value next to SCB was 0.66, indicating that the total of all the variables explained 66% of the value of SCB. Therefore, the assumption that SA and SL affect SCB was reasonable. The empirical results showed that the standardized path coefficient of SL and SA was 0.82, and *P* was less than 0.001, indicating that hypothesis H1 was verified. Hypothesis H2 assumed that employees’ SA has a significant, positive effect on their SCB. The empirical results showed that the standardized path coefficient between SA and SCB was 0.457, and *P* was less than 0.001, indicating that hypothesis H2 was valid as well. Hypothesis H3 described that leadership has a significant and positive effect on employee SCB, which was further verified, as the standardized path coefficient between SL and SCB was 0.395 and *P* was less than 0.001, indicating that H3 was valid [64].

As depicted in Table 6, the influence coefficients of SL on leadership behavior (SL1), safety concerns (SL2) and security control (SL3) were 0.875, 0.937 and 0.894, respectively, and they were all significant at the 0.001 level (*P* < 0.001). The highest explanation degree of SL2 was 0.88, which indicated that the safety of leadership was mainly reflected in SL2. However, it does not mean that SL1 and SL3 are not important. The interpreted value of SL for them also reached 0.77 and 0.80 with high levels of significance. In addition, it showed that SL1, SL2 and SL3 can accurately reflect the safety performance of leaders. The influence coefficients of SA on safety awareness (SA1), safety behavior tendency (SA2) and safety emotion (SA3) were 0.741, 0.826 and 0.836 with high significance (*P* < 0.001). The impacts on SA2 and SA3 were almost the same, namely 0.68 and 0.70, respectively. The SA of employees was mainly reflected in SA2 and SA3. The influence coefficients of SCB on mutual aid among workers (SB1), relationship between superior and subordinate (SB2), participation in suggestion-making (SB3) and self-control (SB4) were 0.938, 0.927, 0.806 and 0.836, with considerable significance (*P* < 0.001). The interpretation amounts of SB1 and SB2 were above 0.8, and that of SB3 and SB4 were 0.65 and 0.56. This showed that SCB can be significantly affected by SB1 and SB2.

## 4. Discussion

The purpose of this paper was to learn the influence between SL, SA and SCB, so as to find a way to improve employee SCB in the railway industry. Based on 238 valid questionnaires obtained in the railway marshalling station, a structural equation model was constructed to analyze the correlation among safety factors. Reliability and validity tests were conducted using Cronbach’s alpha and confirmatory factor analysis (CFA), and hypothesis tests were carried out using SEM. Results showed that SL has a positive effect on SA, and SA has a positive effect on SCB. Further results can be obtained from the correlation coefficients in Table 7.

From the correlation coefficients in Table 7, it was observed that the correlation between SB1 and the manifest dimensions in SA and SL was more than 0.6; the correlation coefficient between SB2 and the manifest dimensions in SA and SL was greater than 0.6, except for SA1 (0.537) and SA3 (0.598); the correlation between SB3 and the manifest dimensions in SA and SL was between 0.5 and 0.6, except for SA1 (0.466); the correlation between SB4 and the manifest dimensions in SL was between 0.5 and 0.6; and correlation between SB4 and the manifest dimensions in SA was between 0.4 and 0.5. These data show that improvement of SB1 and SB2 can directly rely on SA and SL; improvement of SB3 can mainly rely on SA and SL; and improvement of SB4 can partially rely on SL and SA.

Leaders’ attention to safety, behaviors and measures taken for safety issues can significantly affect employees’ attitudes and views on safety, which can further transform into their own behavior and SCB [32]. SL can partially affect SCB of employees [65]. Most of the successful impact of leadership on employee SCB is due to learning through frequent meetings [66]. Through interviews, it was found that employees’ opinions and attitudes towards safety issues are often derived from the imitation and inheritance of leaders, rather than thinking deeply about whether the attitudes held by leaders are correct. However, employees are not willing to voluntarily pander to and abide by the SA and codes of safety conduct of leaders by recognition and understanding. In contrast, their obedience highly relies on rewards and punishments in terms of their behaviors [67]. Also, it was found that employees usually pay attention to their own actions instead of caring about the potential hazards of other people. In addition, they usually do not initiatively help colleagues to stay away from hazards. What is more, out of rebellion, some employees will deliberately break the rules, which will lead to the occurrence of safety accidents [68]. Therefore, in addition to considering the influence of SL, a sound supervision mechanism should be established within the organization so as to achieve more standardized safety management of railway workers [69].

## 5. Conclusions and Implication

In this research, the influencing factors of railway employee SCB are identified. Both SL and railway employee SA significantly promote SCB. SL can significantly affect the SA of railway employees. This is in line with another research of our team. Meng [38] focuses on the study of the relationship between safety awareness and SCB. The results show that safety awareness and SCB are positively correlated, and a simple and far regression model is established to further analyze the correlation between the two concepts. Zhang [70] studied the impact of social security capital on SCB and the intermediary role of independent security motivation, focusing on the process of internal psychological change brought by external changes; this is very meaningful. In particular, this study investigated railway employees of a railway station in China, whose stable management structure provided convenience for this study to ensure there were no great changes during the study period.

### 5.1. Theoretical Implications and Practical Implications

This study proposed improving SCB of railway workers in China and fills a research gap in railway safety by analyzing the influence mechanisms between SA, SL and SCB. Both SL and SA can significantly promote the SCB of railway workers. SL can significantly affect the SA of railway employees. The impacts of leadership and attitudes of employees on SCB were also discussed, which also provided some useful information for safe management of railway employees. In addition, this research has some practical implications and provides a reference for how to promote different types of SCB in the railway industry. Employee attitudes and views on safety issues are more affected by leadership. The behavior of a leader does not effectively guide the behavior of employees. The behavior of employees is mainly dominated by their own ideas. Therefore, managers should pay attention to effectively improving employee attitudes, forming a unified safety regulation, and then encouraging employees to take certain actions voluntarily.

### 5.2. Research Limitations and Future Directions 

However, this study has certain limitations. First, data used in the analysis came from a questionnaire survey, and the source of the data was limited (workers from single railway station). Therefore, it is recommended to use multiple data sources in the future to reduce bias in the data sample. Second, the three postulated hypotheses mainly relied on individual factors in relation to safety, while organizational factors (safety policies, training, reporting, hazard control, working hours, scheduling, staffing, etc.) were neglected. Further research should pay more attention to the influence of organizational factors on safety.

## Figures and Tables

**Table 1:** Dimension division and abbreviations of safety leadership, safety attitude and safety citizenship behavior.

Latent Variable	Dimension	Abbreviations
Safety leadership (SL)	Leadership behavior	SL1
	Safety concerns	SL2
	Security control	SL3
Safety attitude (SA)	Safety awareness	SA1
	Safety behavior tendency	SA2
	Safety emotion	SA3
Safe citizenship behavior (SCB)	Mutual aid among workers	SB1
	Relationship between superior and subordinate	SB2
	Participation in suggestion-making.	SB3
	Self-control	SB4

**Table 2:** Results of reliability analysis of each dimension of the questionnaire.

Variable	Test Item	Item Deleted Scale Mean	Scale Variance Value of Item Deleted	Corrected Item Total Correlation (CITC)	Cronbach’s Alpha Value of Item Deleted	Cronbach’s Alpha
SL	SL1	2.709	1.529	0.828	0.913	0.926
	SL2	2.667	1.260	0.889	0.862	
	SL3	2.675	1.369	0.842	0.899	
SCB	SB1	4.715	5.111	0.839	0.872	0.902
	SB2	4.588	4.674	0.837	0.858	
	SB3	4.470	4.045	0.802	0.868	
	SB4	4.529	3.997	0.746	0.898	
SA	SA1	3.021	3.076	0.674	0.813	0.842
	SA2	2.979	2.637	0.726	0.765	
	SA3	2.933	2.865	0.727	0.762	

Note: SL is safety leadership; SA is safety attitude; SCB is safety citizenship behavior. SL1 is leadership behavior; SL2 is safety concerns; SL3 is security control; SA1 is safety awareness; SA2 is safety behavior tendency; SA3 is safety emotion; SB1 is mutual aid among workers; SB2 is relationship between superior and subordinate; SB3 is participation in suggestion-making; SB4 is self-control.

**Table 3:** Parameter significance estimation and convergence validity of the measurement model.

Construct	Item	Significance Estimation				Factor Load	Topic Reliability	Convergent Validity	Composite Reliability
		Un-std	S.E.	*t*-Value	*P*	Std	SMC	AVE	CR
SL	SL1	1.00				0.87	0.75	0.81	0.93
	SL2	1.13	0.06	18.59	***	0.88	0.78		
	SL3	1.29	0.06	20.60	***	0.96	0.91		
SA	SA1	1.00				0.75	0.56	0.64	0.84
	SA2	1.24	0.11	11.42	***	0.83	0.69		
	SA3	1.15	0.10	11.42	***	0.83	0.69		
SCB	SB1	1.00				0.93	0.86	0.74	0.92
	SB2	1.21	0.05	24.43	***	0.94	0.88		
	SB3	1.32	0.08	17.58	***	0.81	0.66		
	SB4	1.29	0.09	14.93	***	0.74	0.55		

Note: SL is safety leadership; SA is safety attitude; SCB is safety citizenship behavior. SL1 is leadership behavior; SL2 is safety concerns; SL3 is security control; SA1 is safety awareness; SA2 is safety behavior tendency; SA3 is safety emotion; SB1 is mutual aid among workers; SB2 is relationship between superior and subordinate; SB3 is participation in suggestion-making; SB4 is self-control. Un-std is unstandardized estimate. S.E. is standard error. *t*-value is the value of t test. *P* is significant. *** At the 0.001 level, the output is significant. Std is standardized estimate. SMC is squared multiple correlations. AVE is average variance extraction. CR is composite reliability.

**Table 4:** The testing results of discriminant validity among SL, SA and SCB.

	SL	SCB	SA
SL	0.90	-	-
SCB	0.77	0.86	-
SA	0.82	0.78	0.80

Note: SL is safety leadership; SA is safety attitude; SCB is safety citizenship behavior. 0.90, 0.86, 0.80 were the square root of average variance extraction (AVE) of SL, SCB and SA, respectively. 0.77, 0.82, 0.78 were standardized correlation coefficients for SCB and SL, SA and SL, SA and SCB, respectively.

**Table 5:** Model fitting index for SA, SL and SCB measurement models.

χ^2^/df	RMR	GFI	IFI	CFI	PGFI	RMSEA
5.545	0.024	0.881	0.932	0.931	0.512	0.0368

Note: χ^2^/df is the chi-square divided by degrees of freedom, which should be less than 5; if it is less than 3, it suggests a better fit of the model. RMR is root-mean-square residual, which needs to be less than 0.05. RMSEA is root-mean-square error of approximation, which needs to be less than 0.05. GFI is goodness-of-fit index, IFI is incremental fit index. CFI is comparative fit index. GFI, IFI and CFI all need to be more than 0.8. PGFI is parsimony goodness-of-fit index, which needs to be more than 0.5.

**Table 6:** Path coefficient and significance of SEM (structural equation model).

Path			Un-std	S.E.	C.R.	P	Std
SA	<---	SL	1.09	0.098	11.126	***	0.82
SCB	<---	SA	0.381	0.087	4.394	***	0.457
SCB	<---	SL	0.438	0.109	4.038	***	0.395
SA1	<---	SA	1				0.741
SA2	<---	SA	1.261	0.101	12.483	***	0.836
SA3	<---	SA	1.151	0.093	12.349	***	0.826
SB1	<---	SCB	1				0.938
SB2	<---	SCB	1.174	0.046	25.548	***	0.927
SB3	<---	SCB	1.295	0.073	17.856	***	0.806
SB4	<---	SCB	1.274	0.083	15.31	***	0.746
SL1	<---	SL	1				0.875
SL2	<---	SL	1.246	0.058	21.399	***	0.937
SL3	<---	SL	1.137	0.058	19.55	***	0.894

Note: SL is safety leadership; SA is safety attitude; SCB is safety citizenship behavior. SL1 is leadership behavior; SL2 is safety concerns; SL3 is security control; SA1 is safety awareness; SA2 is safety behavior tendency; SA3 is safety emotion; SB1 is mutual aid among workers; SB2 is relationship between superior and subordinate; SB3 is participation in suggestion-making; SB4 is self-control. Un-std is unstandardized estimate. S.E. is standard error. *P* is significant. *** At the 0.001 level, the output is significant. Std is standardized estimate. C.R. is critical ratio; it has the same mean of *t*-value.

**Table 7:** Correlations analysis for all significant dimensions of SA, SL and SCB.

	Correlation Coefficient									
	SL1	SL2	SL3	SB1	SB2	SB3	SB4	SA1	SA2	SA3
**SL1**	1									
**SL2**	0.82	1								
**SL3**	0.782	0.837	1							
**SB1**	0.632	0.677	0.645	1						
**SB2**	0.625	0.669	0.638	0.87	1					
**SB3**	0.543	0.581	0.554	0.756	0.747	1				
**SB4**	0.503	0.538	0.513	0.7	0.692	0.601	1			
**SA1**	0.532	0.57	0.543	0.543	0.537	0.466	0.432	1		
**SA2**	0.6	0.642	0.613	0.613	0.605	0.526	0.487	0.62	1	
**SA3**	0.593	0.635	0.606	0.605	0.598	0.52	0.482	0.612	0.691	1

Note: SL1 is leadership behavior; SL2 is safety concerns; SL3 is security control; SA1 is safety awareness; SA2 is safety behavior tendency; SA3 is safety emotion; SB1 is mutual aid among workers; SB2 is relationship between superior and subordinate; SB3 is participation in suggestion-making; SB4 is self-control.

## Appendix A

The original version of this questionnaire was in Chinese and was translated into English for presentation here. All the items were measured using an eight-point Likert scale. All items exhibited verbal anchors of “strongly disagree” and “strongly agree” at points 1 and 8, respectively. Table A1 shows the items of SCB, SL and SA. Table A2 shows the demographic characteristics of respondents.

**Table A1:** Contents of safety citizenship behavior.

Concepts	Items
SCB	I will help new workers get familiar with the working environment.
	I will guide the safety work procedures of new workers.
	I will help other workers to ensure their safe work.
	I will encourage workers to participate in safety exercises and other safety matters.
	I will help the workers learn the rules and regulations of safe work.
	I will help the workers understand the responsibilities and obligations related to safety.
	I will make safety suggestions during work activities.
	I will encourage workers to participate in the discussion of safety issues.
	I will take the initiative to express my views on Security Affairs.
	I will make a work plan from the perspective of safety.
	I will try my best to protect the workers from potential safety hazards.
	I will pay as much attention to the safety of my workmates as possible.
	I will take the initiative to protect the workers from dangerous situations.
	I will report the hidden danger of work safety accidents to the superior in time.
	I will remind other workers to follow the safe work rules.
	I will supervise the new workers to make sure they work safely.
	I’ll stop the workers from breaking the rules.
	I will tell the new workers that it’s unforgivable to violate the safety regulations.
	I will actively learn new safety knowledge.
	I will actively participate in all safety meetings.
	I will actively understand the improvement and update of relevant safety policies and procedures.
	I will actively participate in safety exercises or safety publicity activities.
	I will try to improve the safety regulations.
	I will actively seek more secure ways to cooperate.
	I will try to improve the security policy and regulations.
	I will timely report the unrealistic contents in the safety regulations to the superior and propose improvement measures.
SL	My leader can actively and fairly participate in the investigation and handling of accidents.
	My leader sets an example and follows the safety rules.
	My leaders often remind workers of the importance of safety.
	My leader often explains safety problems to the workers.
	My leaders often involve workers in the formulation and revision of safety work procedures.
	My leader can earnestly implement safety supervision and deal with problems found in time.
	My leader will take the initiative to exchange safety information with the workers.
	My leadership will fairly allocate resources related to security matters.
	My leadership will encourage subordinates to report the hidden dangers of work safety accidents.
	My leaders will take the initiative to care for their subordinates and help them to solve difficulties.
	My leader will give timely feedback on the suggestions and reports of subordinates.
	My leader will always give affirmation to the safety performance of subordinates.
	My leadership will strictly require subordinates to complete safety objectives and tasks.
	My leader will give timely feedback on the safety performance of subordinates.
	My leader will often praise the safety behaviors of subordinates.
	My leader will correct the hidden danger and criticize the education in time.
	My leadership will strictly require subordinates to abide by the relevant safety management system.
SA	I think on-site work is highly dangerous.
	I think wearing safety shoes and helmets when entering the plant will help prevent accidents.
	I think the safety measures stipulated by the company will reduce my work efficiency.
	For safety, I will know how to use the protective equipment carefully and wear it at any time.
	I always clean and tidy up the things that hinder safety in the workplace.
	Colleagues will pay attention to the safety of work on site and remind me to follow the safety regulations.
	My colleagues will be happy to accept my advice on work safety.
	I will pay attention to the work safety of colleagues or on-site workers and remind them to follow the safety regulations.
	Direct leaders often pay attention to whether employees’ working attitude is lazy.
	When employees are in poor health, the immediate supervisor will immediately stop them from continuing to work.
	Direct leaders often patrol, and will immediately stop employees from taking dangerous work methods.
	The leader will give warning to the employees who still violate the regulations or have unsafe behaviors after repeated admonition.
	Accidents at work are mostly due to bad luck.
	Too much attention to safety procedures will affect work efficiency.
	When the work is too heavy, the safety of the work will be ignored.
	Providing enough safety training can help reduce accidents.
	Pay attention to employees’ work experience, which is helpful to employees’ work performance.
	If employees can participate in training courses, they can effectively improve safety performance.
	Provide a safe working environment for better performance.

**Table A2:** Demographic characteristics of respondents.

Measure	Items	Frequency	Percent
Gender	Male	207	87.0
	Female	31	13.0
Total		238	100
Age	<20	7	2.9
	20–30	113	47.5
	30–40	70	29.4
	40–50	31	13.0
	≥50	17	7.1
Total		238	100
Work experience (in years)	<3	43	18.1
	3–5	91	38.2
	5–10	85	35.7
	≥10	19	8.0
Total		238	100
Education	Junior middle school and below	13	5.5
	High school (including secondary school and technical school)	133	55.9
	Junior College	74	31.1
	Undergraduate or above	18	7.6
Total		238	100
